# Tele-stroke: a strategy to improve acute stroke care in low- and middle-income countries

**DOI:** 10.1097/MS9.0000000000002187

**Published:** 2024-05-17

**Authors:** Hafiza Qurat Ul Ain, Muhammad Junaid Tahir, Khabab Abbasher Hussien Mohamed Ahmed, Faizan Ahmed, Mustafa Mohamed Ibrahim Ali, Esraa Hassan Salih Elhaj, Ghassan E. Mustafa, Areeba Ahsan, Zohaib Yousaf

**Affiliations:** a CMH Multan Institute of Medical Sciences, Multan; b Shaukat Khanum Memorial Cancer Hospital and Research Centre; c Lahore General Hospital, Lahore; d Foundation University School of Health Sciences, Islamabad, Pakistan; e Faculty of Medicine, University of Khartoum, Khartoum, Sudan; f Tower Health, Reading, PA, USA

Stroke represents a major global health concern and is considered the second most common cause of mortality worldwide. Additionally, it poses a significant public health challenge, particularly in low- and middle-income countries (LMICs). The burden of stroke is disproportionate in most parts of Africa^1^ and other resource-poor regions where health systems are underdeveloped and overwhelmed in comparison to high-income countries (HICs). While HICs had an ~42% reduction in stroke burden over the past four decades, the burden in the LMICs has exponentially increased. Stroke-related mortality is much higher in LMICs, with 49 deaths per 100 000 compared to HICs (22 deaths per 100 000). The INTER-STROKE study concluded that stroke patients in LMICs often have suboptimal stroke care due to limited access to services, leading to lower survival rates than HICs^1^.

Telehealth programs have been demonstrated to help decrease the burden among many patients with different diseases. For example, heart failure patients who have been discharged from the hospital benefit from continuous monitoring and psychological support, which has been shown to improve their symptoms and compliance with medications^2^. Patients with type 2 diabetes who use telehealth have shown a decrease in their HbA1c compared to those who do not^3^. Additionally, a study conducted among adolescent patients with inflammatory bowel syndrome showed fewer in-office visits and demonstrated a positive impact on their quality of life after using telemedicine programs^4^. Telehealth has also proven its effectiveness in managing postpartum depression and associated maternal mental health problems in the postpartum period^5^.

Generally, Tele-neurology is the wide-ranging use of telemedicine for both acute and outpatient neurology care. Tele-stroke on the other hand is a subset of Tele-neurology^6^.

Tele-stroke is a method of identifying, assessing, treating, and monitoring stroke patients remotely using the internet or telephone technology^1^. Tele-stroke programs aim to increase access and prompt care for stroke patients through live video consultations between clinicians at the site where the patient presents (the originating site) and stroke specialists at a distant site^7^. Without such consultation, physicians in the emergency department may be hesitant to offer thrombolytic therapy for acute ischaemic stroke. Tele-stroke can reduce “door-to-needle time” and stroke-related morbidity and mortality cost-effectively. Increasing access to specialized stroke care through Tele-stroke is a promising development for rural areas, where stroke mortality is about 20% higher than in urban areas, which can be attributed to the scarcity of specialized stroke teams in rural hospitals. Thus, Tele-stroke services fill this gap and allow rural hospitals to provide adequate care in comparison to hospitals with in-person stroke specialists^7^.

Additionally, Tele-stroke services can support existing stroke services. It is also worth noting that currently, telemedicine is being explored to improve access to healthcare in LMICs including areas of conflict^8^.

Furthermore, Tele-stroke has improved access to stroke care, such as thrombolysis, leading to better patient outcomes^1^. Rehabilitation after a stroke requires the input of several skilled health personnel, including physiatrists, physiotherapists, speech therapists, and occupational therapists^9^. Home-based telerehabilitation can help meet the needs of stroke survivors in resource-limited rural settings^9^. Governments in LMICs should employ telehealth tools to improve their healthcare systems^10^. Telemedicine, particularly tele-stroke programs, is a critical technique for linking rural areas to expert stroke care. Tele-stroke enables remote consultations with physicians, enabling quicker decisions^11^.

On the other hand, the cost-effectiveness of tele-stroke services is a major issue for governments seeking to maximize resource utilization and improve healthcare outcomes. Tele-stroke services have shown promising advantages in enhancing resource utilization and lowering overall healthcare costs by using technology to provide remote consultations^1,7^. The reduced “door-to-needle time” achieved by tele-stroke therapy leads to improved patient outcomes, potentially lowering the long-term economic burden associated with stroke-related disability^9^. Furthermore, tele-stroke services’ capacity to connect specialists with remote places reduces the need for patient transfer, minimizing transportation costs and increasing healthcare delivery efficiency. Policymakers should prioritize rigorous cost-benefit analyses to assess the economic sustainability and long-term financial benefits of tele-stroke services, ensuring a judicious allocation of resources to improve stroke therapy in low- and middle-income nations^10^.

Owing to the difficulty in dealing with stroke victims, the necessity of the first contact doctor or healthcare worker is highlighted in rural healthcare settings. The ability of these primary healthcare practitioners to diagnose and treat stroke symptoms becomes critical for timely intervention. Their skills and knowledge have an immediate impact on the accuracy of tests, diagnoses, and the execution of appropriate treatment^12^. Another necessity is establishing networks of stroke care professionals and the government’s use of telehealth capabilities to enhance healthcare systems^13^.

India proposes a successful model for the implementation of tele-stroke. In March 2014, a tele-stroke program was launched in the state of Himachal Pradesh, which lies in Northern India. This area consists of mountainous terrain and landscape and has a population of more than 6 million. 90% of the population lives in villages. There were 2 tertiary care hospitals with 2 neurologists in each hospital. These circumstances constituted a problem in delivering stroke care to patients. To tackle this issue, the tele-stroke program connected government hospitals with computed tomography (CT) scan facilities to the two tertiary care centres. There were 17 district hospitals, and they were divided between the two centres for consultations^13^. The program also trained medical officers in district hospitals to identify stroke and interpret CT scans without contrast. Training also contained written protocols for thrombolysis to be followed by the trainees. The program also provided free-of-charge tPA at all centres to all patients. Regarding means of communication between doctors in the district hospitals and the neurologists in the tertiary centres, accessible means of communication such as WhatsApp were used^13^.

Through the course of the following year and by May 2015, the tele-stroke program had achieved the result of providing thrombolysis for a total of 26 patients at the district centres which were labelled primary stroke centres. Of the 26 patients, 8 were female and 18 were male with an overall age ranging from 26 to 80 years old^13^. There were no in-hospital mortalities. Of the 26 patients, only 2 suffered intracranial haemorrhage after thrombolysis and both cases were nonfatal. These results were compared to the results from Indira Ghandi Medical College (IGMC), which is the largest tertiary care centre in the Himachal Pradesh area. It was found that IGMC provided thrombolysis for 22 patients during the same period proving the effectiveness of the tele-stroke program^13^.

It is also worth noting that within one month of starting the program in March 2014, a patient was offered thrombolysis in a district hospital. This is noteworthy because it was the first time a patient was thrombolysed in a district hospital in the state without the presence of a neurologist in person^13^. This patient was a 75-year-old male. He presented within 40 min of developing right-sided weakness with the right upper limb power grade 1/5 and the right lower limb power grade 3/5. TPA was administered after the exclusion of haemorrhage. Door-to-needle time was 40 min. On discharge, the power grade in the right upper limb was 3/5 and 5/5 in the right lower limb marking the success of management^13^.

However, despite the ability of tele-stroke to improve access to specialized stroke care, there are significant barriers to implementation, such as physician inertia and a lack of infrastructure including inadequately equipped urgent care facilities, limited availability of reliable brain imaging, deficient access to stroke units and remote stroke services, a scarcity of specialized healthcare providers such as neurologists, neurosurgeons, physiotherapists, occupational therapists, speech therapists, and nurses, as well as limited access to health insurance^14^.

Despite disparities in wealth, education, basic health indicators, and funding of healthcare expenses in LMICs, stroke care services can be improved with remodelling and reallocation of resources^14^. Evidence-based recommendations to improve acute stroke care in LMICs include the provision of emergency medical transportation services, scaling up acute stroke care and interventions, improving access to brain imaging services, the development of a human resource capacity strategy for acute stroke care, the centralization of stroke services, telecare, the establishment of stroke registries, and funding for stroke care^1^.

Furthermore, to tackle these obstacles hindering tele-stroke application in LMICs, it’s a necessity to study successful models such as the program in Himachal Pradesh. LMICs can strive to overcome infrastructure deficiencies by adding tele-stroke to established systems through the utilization of pre-existing services. Such as connecting rural hospitals to tertiary care centres. Another example of this is the use of accessible means of communication for consultations such as WhatsApp or other social media platforms^13^. Another approach would be to use mobile phones (phone calls) for communication^13,15^. If possible, governments can directly improve deficits to facilitate the implementation of tele-stroke by providing means of communication such as high-quality internet and suitable devices[6].

It is also of significant importance to train medical personnel in rural areas as they need to be qualified to identify stroke, interpret imaging, and provide the proper management^13^. These doctors not only need to be trained but also need to be encouraged to take part in the program. This can be achieved through the organization of workshops for medical officers to train in the skills needed. To keep medical officers motivated to be part of tele-stroke, a reward system can be implemented. In addition, keeping the data of patients who were managed in rural hospitals can also help as a reminder of the impact of their work.

Funding is crucial to ensure the success and sustainability of the program, as the provision of tPA in rural areas is a priority. It is also required for the provision of training workshops for medical officers as well. This funding can come from governments, donations from NGOs, the UN or from the community.

Governments should construct adequate telecommunications networks and provide technical and financial assistance for remote acute stroke therapy. Essentially, improving stroke therapy in distant areas necessitates empowering healthcare staff at the forefront, using telemedicine, and implementing systemic policy changes^16^.

In conclusion, Tele-stroke offers significant benefits to both patients and doctors. Patients gain convenient access to medical care from their homes, saving time and money on travel while accessing neurologists regardless of geographical constraints. Additionally, tele-stroke promotes cost savings for patients and healthcare facilities alike. Improved continuity of care is facilitated through better communication between providers, enabling remote follow-up consultations and monitoring. Patients are more engaged in managing their health, leading to better outcomes, while doctors can see more patients efficiently. Tele-stroke also reduces the risk of exposure to infectious diseases by minimizing the need for in-person visits, particularly crucial during health crises. Overall, tele-stroke enhances access, efficiency, and patient outcomes, making it a valuable tool in modern healthcare delivery.

## The process of tele-stroke care

### Technological components

In order to enable smooth communication and data transfer between healthcare professionals and stroke experts, tele-stroke treatment depends on an advanced infrastructure made up of several technology components^11^. Fundamentally, tele-stroke systems usually include high-definition video conferencing features that allow distant stroke specialists to evaluate patients visually in real-time. Furthermore, sophisticated imaging technologies like CT and MRI scans are essential to the diagnosis process because they enable precise assessment of the severity of strokes and suitable treatment planning. Furthermore, to facilitate thorough patient care and remote vital sign monitoring, tele-stroke systems frequently incorporate electronic medical records (EMRs) and telemonitoring equipment^17^. It has the potential to be revolutionary to apply artificial intelligence and machine learning to support tele-stroke care. This includes a three-month prognosis and the ability to forecast the duration of the hospital stay in addition to a quicker and more accurate diagnosis using imaging analysis. By examining patient records, intelligent electronic medical records (EMRs) may aid with decision-making and do free text searches. Speech recognition has developed to the point where it is dependable and incredibly practical. Contextual awareness in communication and alert systems can boost patient flow efficiency and lead to better results^18^. One of the most recent developments in medical record keeping is intelligent EMR. A complete EMR is being read by AI using a method known as natural language processing (NLP). Natural language processing (NLP) facilitates the transformation of spoken language into a structured representation that allows information to be automatically identified and extracted^19^. Neurologists have already utilized NLP to examine unstructured data from EHRs, such as reports from neuroradiology and progress notes. NLP was successfully utilized to categorize patients into subgroups of ischaemic stroke. Because it can analyze prior history for data that might impact care (e.g. atrial fibrillation or recent surgery), inform critical lab findings, and compute scoring for various stroke scales using defined factors, intelligent electronic medical records (EMRs) can also be a significant tool for tele-stroke^20^.

### Protocols

Standardized procedural guidelines are followed in the tele-stroke care process to guarantee that patients receive stroke treatment in an effective and timely manner. Tele-stroke practices may involve more extensive use of telemedicine across the entire continuum of stroke care, with some even consulting on all neurologic emergencies. These guidelines include advice for operations, management, administration, and economics and outline a network of computer systems and audiovisual communication for the delivery of tele-stroke therapeutic services. These interactive interactions provide access to knowledge, improve clinical practice, and improve quality outcomes and metrics by connecting patients with acute ischaemic and haemorrhagic stroke syndromes with acute care institutions and remote and on-site healthcare practitioners. These recommendations do not dictate or suggest general clinical protocols for the management of stroke patients; rather, they are particular to tele-stroke services^21^. During the session, the stroke specialist does a comprehensive neurological examination via video conference, which includes assessing symptoms, doing neurological tests, reviewing pertinent medical history, and reviewing imaging results. The foundation for diagnosing individuals with a potential stroke and selecting the best course of therapy is provided by the clinical findings and history. It may be possible to increase diagnostic accuracy and reliability by concentrating on three findings: aberrant speech, arm drift, or acute facial paresis^22^.

### Patient experiences

Due to the distance to specialist neurologic services, stroke patients in rural areas may not receive the same level of treatment or diagnosis as hospitalized patients. The effective technique of tele-stroke consultation allows for prompt diagnostic and therapy recommendations for stroke victims. Tele-stroke consultations save patients from underserved rural areas from having to make lengthy trips to far-off medical facilities, which can delay treatment and worsen results. The tele-stroke strategy also encourages patients to take an active role in managing their illness by providing them with individualized treatment from diverse teams of medical specialists^23^.

The authors offer the following recommendations:

1. Establish transcontinental, inter-regional, intra-regional, and national networks of stroke care specialists to improve the reach of acute stroke care. This can be done by utilizing pre-existing accessible means of communication like WhatsApp and other already widespread instant messaging applications. Another option can be a government-funded program for developing a dedicated platform by contacting application developers to develop a dedicated contact platform for tele-stroke consultations. However, this module requires a designated fund.
2. Integrate tele-stroke practices into stroke service systems. According to a study carried out in Southeast Bavaria, Germany, the Telemedical Project for Integrative Stroke Care (TEMPiS) has demonstrated its ability to enhance acute specialised stroke care through the establishment of dedicated stroke wards, continuous quality management, and round-the-clock telemedicine access to neurological experts which include thrombolysis services^24^.
3. Establish a suitable telecommunications platform that transmits images at a reasonable speed and considers immediate technical assistance and funding for the remote management of acute stroke. The use of already available, easily accessible communication channels, such as WhatsApp and other popular instant messaging apps, can accomplish this. A government-funded initiative to create a specific platform for tele-stroke consultations might be an additional choice. Application developers could be approached to create this platform. This module, however, needs a specific fund.
4. Funding for the remote management of acute stroke. In a study done in the USA tele-stroke has demonstrated cost-saving benefits by enabling the treatment of a greater number of patients with IV tPA and increasing the number of discharges straight to the patient’s home, hence minimizing the need for long-term rehabilitation stays and saving up to $44,000 yearly per hospital in the network. However, since existing compensation for telemedicine is restricted by law to specific specialities and low-access locations, cost-sharing programs or assistance from the state and federal levels are required to make telemedicine networks commercially viable^25^.

## Ethical approval

Ethical approval was not required for this type of manuscript.

## Consent

Consents were not required for this type of manuscript.

## Source of funding

No financial support was acquired for this article.

## Author contribution

H.Q.U.A., M.J.T. and K.A.H.M.A. conceived the idea, contributed equally to this manuscript and shared the first author position. H.Q.U.A., M.J.T., K.A.H.M.A., F.A., M.M.I.A., E.H.S.E., A.A. and Z.Y. performed a literature review and wrote the manuscript. G.E.M., K.A.H.M.A., M.M.I.A., E.H.S.E., Z.Y. and M.J.T. reviewed the manuscript and critically revised it to the final form. All authors approved the final version of the manuscript.

## Conflicts of interest disclosure

The authors declare no conflict of interest.

## Research registration unique identifying number (UIN)

Not applicable because it is not needed for this type of study.

## Guarantor

Khabab Abbasher Hussien Mohamed Ahmed.

## Data availability statement

The data used in this report are available from the corresponding author upon reasonable request.

## Provenance and peer review

Not commissioned, externally peer-reviewed.
